# Supplementary material for: Identifying Eigen-like hydrated protons at negatively charged interfaces
Source: Nat Commun. 2020 Jan 24;11:493. doi: 10.1038/s41467-020-14370-5 (PMC6981112; doi:10.1038/s41467-020-14370-5)
Supplement: Supplementary file 1 — Supplementary Information [file 41467_2020_14370_MOESM1_ESM.pdf]

Supplementary Information

Identifying Eigen-like hydrated protons at negatively charged interfaces

Tyrode et al.

Supplementary Note 1. Surface pressure vs molecular area isotherms for deuterated arachidic acid (dAA) and sodium eicosyl sulphate.

The surface pressure vs molecular area isotherm of a deuterated arachidic acid monolayer deposited on 1 μM NaCl solution is shown in Supplementary Fig. 1a. At molecular areas >~23 $Å^2$ the isotherm shows a coexistence region between the 2D gas phase and a 2D tilted condensed phase. The pure tilted condensed phase then extends between ~23 $Å^2$ and ~19 $Å^2$, where the abrupt change in slope at ~27 mN m^{-1} marks the transition to the untilted condensed phase. At natural pH the isotherm has been previously shown to be largely insensitive to the presence of monovalent ions in solution up to concentrations as high as 1 M.[1] This despite the fact that the monolayer partially deprotonates upon addition of NaCl to the subphase.[1, 2] The measurements presented in the article were carried out at constant surface pressure of 20 mN m^{-1}. The dAA monolayer is then found in the tilted condensed phase with an average area per molecule of ~20 $Å^2$.

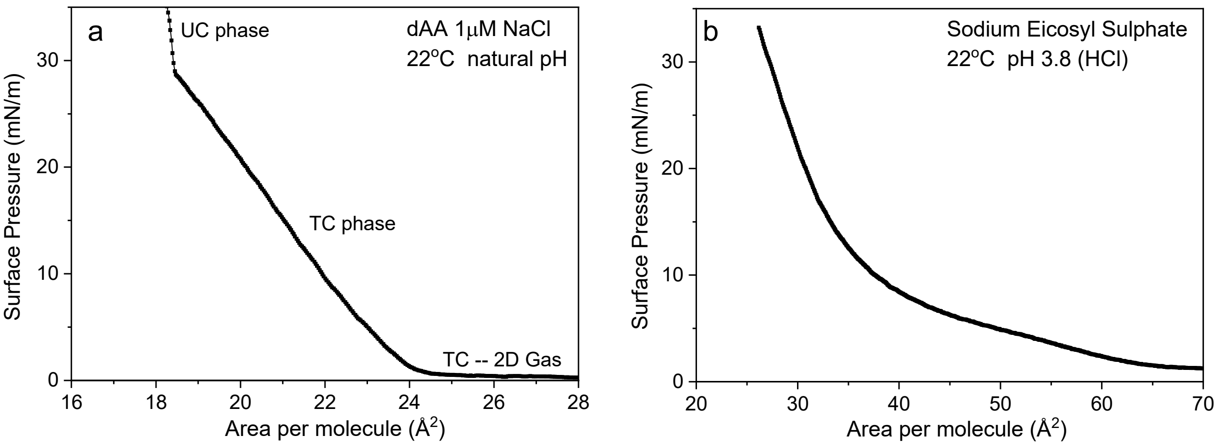

Supplementary Figure 1. Langmuir compression isotherms of (a) a deuterated arachidic acid monolayer on a 1mM NaCl subphase at pH 5.8, and (b) sodium eicosyl sulphate on a pH 3.8 subphase prepared with HCl. For dAA, the untilted condensed (UC), tilted condensed (TC) phase, as well as the TC – 2D gas coexistence regions are labelled in the figure. Temperature 22 ± 0.2 °C.

The compression isotherm for the fatty sulphate monolayer on a HCl solution at pH 3.8 is shown in Supplementary Fig. 1b. At such conditions, this highly charged monolayer is found in a liquid expanded phase and is significantly less stable than the dAA fatty acid. The eicosyl sulphate is slightly soluble when fully charged and tends to desorb from the surface as a function of time.[3] Additionally, the fatty sulphate can hydrolyse to form eicosanol, a process that is accelerated at lower pH.[3, 4] These elements, together with other potential impurities found in the parent compound, may obscure a phase transition[3] in the isotherm that could be triggered by the binding of sodium cations to the sulphate headgroup. The VSF measurement presented in Figure 4 of the main article was carried out at a surface pressure of 2.5 mN m^{-1}, (corresponding roughly to an area per molecule of 65 $Å^2$) to reduce the possibility of such a binding event. Moreover, the fatty sulphate has an intrinsically lower pKa that the fatty acid (~0 compared to ~5), which implies that it is significantly more deprotonated, even at pH 3.8.

Supplementary Note 2. Description of the orientational analysis for the antisymmetric stretch of H_3O^+.

The theoretical framework for calculating the orientation of specific bonds of molecules at the interface from polarized VSF spectra is well-established.[5, 6, 7, 8, 9] Experimentally determined intensity or amplitude ratios obtained under the different polarization combinations are compared with theoretical curves that model the expected changes in relative intensities as a function of the orientation of the group under study. The first step is to identify the independent components of the hyperpolarizability tensor $\beta^{(2)}$ from considerations of molecular symmetry. The H_3O^+ of the Eigen core can be treated as belonging to the C_{3v} point group, for which the orientation is best described in terms of the tilt (θ) of the principal axis of symmetry (C_3) relative to the surface normal. For the degenerate antisymmetric stretch, only four non-zero elements of the hyperpolarizability remain, of which all are equivalent: $\beta^{(2)}_{aca} = \beta^{(2)}_{caa} = \beta^{(2)}_{bcb} = \beta^{(2)}_{cbb}$. These hyperpolarizability $\beta^{(2)}$ tensor elements are then transformed from molecular coordinates to laboratory coordinates using an Euler rotation transformation matrix, and then orientationally averaged to obtain the susceptibility tensor $\chi^{(2)}$. The expression for $\chi^{(2)}$ can be integrated over the azimuthal and twist angles, leaving a final expression of $\chi^{(2)}$ as a function of the tilt angle (θ) and the non-zero $\beta^{(2)}$ elements. Multiplication by the appropriate Fresnel factors then gives the theoretical SF intensity as a function of orientation parameters, shown in Figure 3b of the article.

The hyperpolarizability elements for the stretching vibrations of the H_3O^+ Eigen core are not readily available. However, for the degenerate antisymmetric stretch, since there is only one independent element, the four non-zero tensor elements can be set to 1. On the other hand, for the orientation analysis of the symmetric stretch, there are two independent elements: $\beta^{(2)}_{aac} = \beta^{(2)}_{bbc}$; $\beta^{(2)}_{ccc}$. Consequently, the theoretical curves for the symmetric stretch cannot be plotted with confidence, but the general trends are expected to be similar for those reported for other C_{3v} symmetry vibrations.[8, 9]

Supplementary References

1. Sthoer A, Hladilkova J, Lund M, Tyrode E. Molecular insight into carboxylic acid - alkali metal cations interactions: reversed affinities and ion-pair formation revealed by non-linear optics and simulations. *Phys Chem Chem Phys* **21**, 11329-11344 (2019).

2. Tyrode E, Corkery R. Charging of Carboxylic Acid Monolayers with Monovalent Ions at Low Ionic Strengths: Molecular Insight Revealed by Vibrational Sum Frequency Spectroscopy. *J Phys Chem C* **122**, 28775-28786 (2018).

3. Wüstneck R, Siegel S, Ebisch T, Miller R. Surface Behavior of Spread Sodium Eicosanyl Sulfate Monolayers.: 1. π/AIsotherms Determined on a Langmuir Film Balance and on Drop Surfaces and Brewster Angle Measurements. *J Colloid Interface Sci* **203**, 83-89 (1998).

4. Wüstneck R, Siegel S, Ebisch T, Miller R. Surface Behavior of Spread Sodium Eicosanyl Sulfate Monolayers.: 3. Eicosanyl Sulfate Monolayers Spread on a NaCl Subphase. *J Colloid Interface Sci* **203**, 90-96 (1998).

5. Hirose C, Akamatsu N, Domen K. Formulas for the analysis of the surface SFG spectrum and transformation coefficients of cartesian SFG tensor components. *Appl Spectrosc* **46**, 1051-1072 (1992).

6. Zhuang X, Miranda PB, Kim D, Shen YR. Mapping molecular orientation and conformation at interfaces by surface nonlinear optics. *Physical Review B: Condensed Matter and Materials Physics* **59**, 12632-12640 (1999).

7. Bell GR, Li ZX, Bain CD, Fischer P, Duffy DC. Monolayers of Hexadecyltrimethylammonium p-Tosylate at the Air-Water Interface. 1. Sum-Frequency Spectroscopy. *J Phys Chem B* **102**, 9461-9472 (1998).

8. Tyrode E, Johnson CM, Baldelli S, Leygraf C, Rutland MW. A Vibrational Sum Frequency Spectroscopy Study of the Liquid-Gas Interface of Acetic Acid-Water Mixtures: 2. Orientation Analysis. *J Phys Chem B* **109**, 329-341 (2005).

9. Wang H-F, Gan W, Lu R, Rao Y, Wu B-H. Quantitative spectral and orientational analysis in surface sum frequency generation vibrational spectroscopy (SFG-VS). *Int Rev Phys Chem* **24**, 191-256 (2005).
